# The *C. elegans* deglutamylase CCPP-6 does not operate redundantly with CCPP-1 in gross cilia integrity

**DOI:** 10.17912/micropub.biology.000740

**Published:** 2023-05-22

**Authors:** Abigail S Klimas, Jessica Dominguez, Bhumi P Shah, Zion Y Lee, Nina Peel

**Affiliations:** 1 Department of Biology, College of New Jersey, Ewing, New Jersey, United States

## Abstract

Tubulin glutamylation is a reversible modification of the microtubules that regulates cilia stability and function. The addition of glutamates to the microtubule is catalyzed by members of the TTLL family of enzymes, while the removal is carried out by a family of cytosolic carboxypeptidase (CCP) enzymes. *C. elegans* has two deglutamylating enzymes, CCPP-1 and CCPP-6 . CCPP-1 is required for ciliary stability and function in the worm, however CCPP-6 is dispensable for cilia integrity. To investigate redundancy between the two deglutamylating enzymes we made a *ccpp-1 ( ok1821 ); ccpp-6 ( ok382 )* double mutant. The double mutant shows normal viability, and the dye-filling phenotypes are not worse than the *ccpp-1* single mutant, suggesting that CCPP-1 and CCPP-6 do not function redundantly in *C. elegans* cilia *.*

**Figure 1. ccpp-1; ccpp-6 double mutant phenotypes are not indicative of redundancy:**
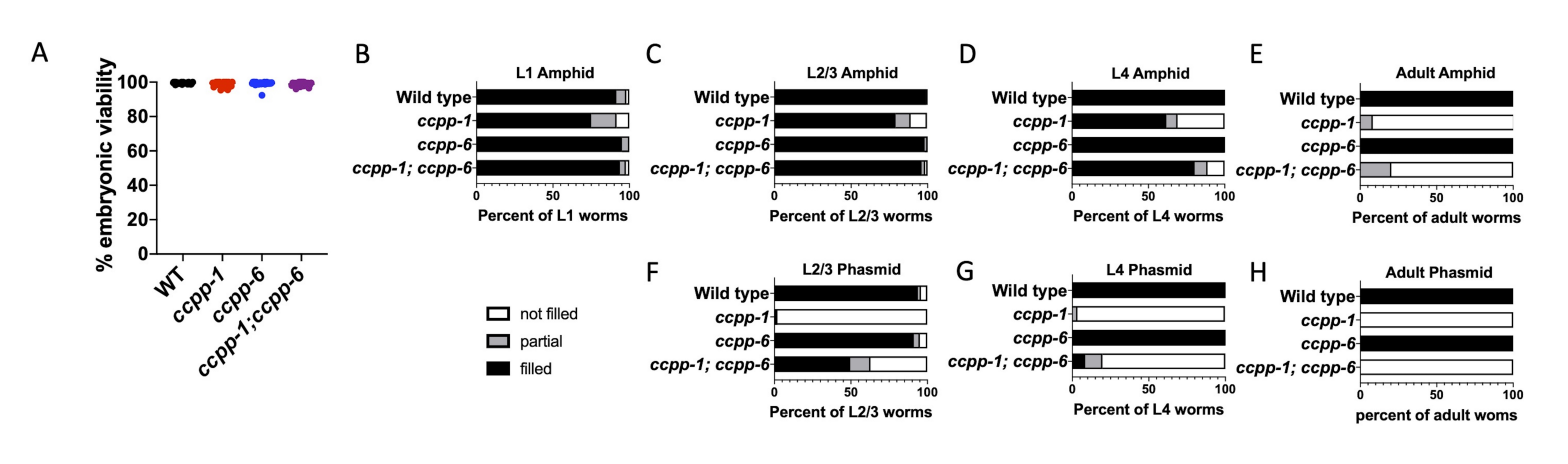
A) Viability of the single and double mutants is comparable to WT. Dye-filling of amphid (B-E) and phasmid (F-H) neurons at L1 (B), L2/3 (~40h post laying; C&F), L4 (D&G), and adult (E&H) life stages. Three (L4 and adult) or four (L1 and L2/3) independent trials were conducted, each trial had an n of >15 worms/genotype (total n: L1 >120; L2/3 >97; L4 >55; adult >63).

## Description

Microtubule glutamylation, the reversible addition of glutamic acid, is abundant within neurons and cilia and required for their function (Janke and Magiera 2020). In *C. elegans* both hyper- and hypo-glutamylation are associated with defects of the neuronal cilia (O’Hagan *et al.* 2011; Chawla *et al.* 2016). The worm genome encodes two deglutamylating enzymes, CCPP-1 and CCPP-6 (Kimura *et al.* 2010). By homology with mouse proteins, CCPP-1 is expected to shorten the polyglutamate chain, while CCPP-6 removes the branchpoint glutamate (Wu *et al.* 2017). Loss of CCPP-1 causes hyperglutamylation and is associated with male mating defects and degeneration of the amphid and phasmid cilia (O’Hagan *et al.* 2011). In contrast, although CCPP-6 promotes regrowth of the PML neuron after injury (Ghosh-Roy *et al.* 2012), a deletion mutation in *ccpp-6* neither impairs dye-filling, nor male mating behavior, indicating that the cilia are largely intact in the absence of CCPP-6 (Dominguez *et al.* 2022). A recent *in vitro* study found that the mammalian homologs of CCPP-1 and CCPP-6 , CCP-1 and CCP-5, synergize for maximal deglutamylation of tubulin (Chen and Roll-Mecak 2023) . This led us to hypothesize that simultaneous loss of CCPP-1 and CCPP-6 may cause a worse phenotype than either single mutant. In addition, because *C. elegans* has only two deglutamylating enzymes it affords a unique opportunity to determine the effects of complete inhibition of deglutamylation. To this end, we made a *ccpp-1 ( ok1821 ); ccpp-6 ( ok382 )* double mutant strain and compare single and double mutant phenotypes.

Mutations in the human homolog of CCPP-1 are associated with infantile-onset neurodegeneration and are frequently fatal (Shashi *et al.* 2018). Embryonic viability defects have not been reported for *ccpp-1* mutants, but we reasoned that subtle defects might be exacerbated, or new phenotypes may arise, in a double mutant. To determine whether loss of deglutamylating activity is associated with embryonic viability defects in *C. elegans* we carried out viability assays for double and single mutants. We did not observe a reduction in viability in either the double or single mutants (Fig1A). Thus the combined loss of both deglutamylating enzymes in *C. elegans* does not impair viability.

Hermaphrodites carrying *ccpp-1* mutations show progressive ciliary degeneration whereby dye-filling is seen in early larval stages, but adult worms almost completely fail to dye-fill (O’Hagan *et al.* 2011). This indicates that CCPP-1 is required for ciliary maintenance, but not formation. One potential explanation for this observation is that CCPP-6 alone might be sufficient to support cilia stability during larval development in the absence of CCPP-1 . We tested this hypothesis by assessing dye-filling in our mutants.

In accordance with published findings, in young *ccpp-1* larvae (L1, Fig 1B; L2/3, Fig 1C) dye uptake of amphid neurons was high, indicative of the presence of grossly normal cilia (O’Hagan *et al.* 2011). At the L4 stage approximately 60% of the *ccpp-1 ( ok1821 )* single mutants showed normal dye-filling of the amphid neurons (Fig 1D) and, consistent with previous reports of a progressive degeneration of cilia, adult worms show a complete absence of normal dye-filling (Fig 1E). The *ccpp-1 ; ccpp-6* double mutants show a similar pattern of age-related decline in amphid dye-filling, that is not present in the *ccpp-6* single mutants. In the phasmid neurons the dye-filling defect manifests at an earlier stage such that *ccpp-1* mutants already show a strong defect at larval stages 2/3 and this continues through adulthood (Fig 1F-H). This phenotype is not worse in the double mutant, and at the L2/3 stage is reproducibly improved (Fig 1F). Our data therefore indicate that in the *ccpp-1 ; ccpp-6* double mutant, the cilia phenotype is not worse than the *ccpp-1* single mutant, moreover it recapitulates the age-dependent Dyf phenotype. While it is unexpected that the double mutant has a slight improvement in the Dyf phenotype, our data nevertheless suggest that redundancy between CCPP-1 and CCPP-6 does not contribute to sensory cilia development in *C elegans* larvae.

In summary, we find that the *ccpp-1 ; ccpp-6* double mutant does not have viability defects and shows similar dye-filling phenotypes to the *ccpp-1* single mutant. Thus neither viability nor *ccpp-1 -* induced cilia dysfunction are worsened by the combined absence of CCPP-6 . This argues against redundancy in function between CCPP-1 and CCPP-6 in the ciliated neurons. Since redundancy between the two deglutamylating enzymes cannot account for the absence of cilia defects during development it suggests a differential requirement for CCPP-1 function in larvae compared with adults.

## Methods

For viability assays, single L4 hermaphrodites were put onto 35mm plates at 20°C. Each worm was transferred to a new plate every 24 hours. Plates were scored after 24h and the number of viable worms and dead embryos was recorded. For dye-filling, to enrich for younger larval stages worms were washed off a plate to leave only embryos, and the plates were incubated at 20°C. Plates were processed for dye-filling either after ~16h (L1 worms) or ~40h (L2/3), or ~72h(L4). Dye-filling assays were carried out as detailed in Chawla *et al.* 2016. Briefly, worms were incubated in 5 μg/ml DiI (1,1′-dioctadecyl-3,3,3′, 3′-tetramethylindocarbocyanine perchlorate) diluted in M9 for 30min. Worms were washed three times in M9 buffer and allowed to crawl on a worm plate for 2h. Worms were anesthetized in 15mM sodium azide, mounted on a 2% agarose pad (diluted in M9), and viewed under fluorescent light on a Nikon E800 with a 20x 0.75 NA objective.

## Reagents

**Table:** 

**Strain name**	**Genotype**	**Available from**
NIN59	*ccpp-6 ( ok382 ) II *	authors
NIN68	*ccpp-1 ( ok1821 ) I; ccpp-6 ( ok382 ) II *	authors
NIN79	*ccpp-1 ( ok1821 ) I *	authors
